# Factors Affecting Time to Sputum Culture Conversion in Adults with Pulmonary Tuberculosis: A Historical Cohort Study without Censored Cases

**DOI:** 10.1371/journal.pone.0142607

**Published:** 2015-11-11

**Authors:** Rie Kanda, Taishi Nagao, Nguyen Van Tho, Emiko Ogawa, Yoshitaka Murakami, Makoto Osawa, Yoshinori Saika, Kenji Doi, Yasutaka Nakano

**Affiliations:** 1 Division of Respiratory Medicine, Department of Medicine, Shiga University of Medical Science, Shiga, Japan; 2 Health Administration Center, Shiga University of Medical Science, Shiga, Japan; 3 Department of Medical Statistics, Shiga University of Medical Science, Shiga, Japan; 4 Division of Infection Control and Prevention, Shiga University of Medical Science Hospital, Shiga, Japan; 5 Department of Radiology, Hirakata Kohsai Hospital, Osaka, Japan; 6 Department of Radiology, Morinomiya Hospital, Osaka, Japan; Fundació Institut d’Investigació en Ciències de la Salut Germans Trias i Pujol, Universitat Autònoma de Barcelona, SPAIN

## Abstract

**Background:**

In patients with pulmonary tuberculosis (TB), shortening the time to sputum culture conversion is desirable to reduce the likelihood of mycobacterial transmission. A persistent positive sputum culture after 2 months of treatment is reported to be associated with the presence of cavitation and the extent of disease on chest X-ray, high colony count, diabetes mellitus, and smoking. However, little is known about factors affecting the time to sputum culture conversion. This study was conducted to evaluate factors affecting the time to sputum culture conversion throughout the course of treatment in adults with pulmonary TB.

**Methods:**

This study was performed using a database of the medical records of patients with active pulmonary TB who were treated at Hirakata Kohsai Hospital in Hirakata City, Osaka, Japan, from October 2000 to October 2002. Cox proportional-hazards analysis was used to evaluate factors affecting the time to sputum culture conversion after adjusting for potential confounders.

**Results:**

The data of 86 patients with pulmonary TB were analyzed. The median time to sputum culture conversion was 39 days, and the maximum time was 116 days. The Cox proportional-hazards analysis showed that a higher smear grading (HR, 0.40; 95%CI, 0.23–0.71) and a history of ever smoking (HR, 0.48; 95%CI, 0.25–0.94) were associated with delayed sputum culture conversion.

**Conclusion:**

High smear grading and smoking prolonged the time to sputum culture conversion in adults with pulmonary TB. To effectively control TB, measures to decrease the cigarette smoking rate should be implemented, in addition to early detection and timely anti-TB treatment.

## Introduction

The estimated incidence of tuberculosis (TB) is declining, but TB is still second only to HIV/AIDS as a single infectious agent responsible for the greatest number of deaths. [1] To prevent TB transmission, patients with infectious TB should be identified early, isolated effectively, and treated appropriately. However, in many countries, patients with infectious TB are not strictly isolated from healthy subjects because of poverty and poor medical systems. Especially under such conditions, the duration during which the patients cough up viable bacilli is a matter of concern with respect to TB elimination or control.

Sputum culture conversion after the first 2 months of treatment is recognized as a surrogate biomarker of long-term cure. [2,3] It has been reported that the presence of cavitation [4], the extent of the disease [5,6] on chest X-ray (CXR), high colony count, [5] diabetes mellitus, [6] and smoking [4,7–9] are associated with culture non-conversion after the first 2 months of treatment. However, no study has evaluated which factors affect the time to sputum culture conversion throughout the course of treatment in a cohort without any censored cases. In addition, most previous studies from industrialized countries were not adequately controlled for confounders.

This study was conducted to evaluate the factors affecting the time to sputum culture conversion throughout the course of treatment in adults with active pulmonary TB after controlling for potential confounders.

## Materials and Methods

### Ethics Statement

This study was conducted using an anonymous database of hospital records that, was not linked to the personal data of individual patients. The Institutional Review Boards of the Shiga University of Medical Science (25–43) and Hirakata Kohsai Hospital approved the study.

### Patients

This historical cohort study was performed using a database of the medical records of 120 patients with pulmonary TB who were admitted to Hirakata Kohsai Hospital (formerly called Keihanna Hospital) in Hirakata City, Osaka, Japan, from October 2000 to October 2002. The prevalence of TB in Osaka has been the highest in Japan for more than 20 years. Inclusion criteria for this study were: (1) adults aged 20–80 years, (2) newly diagnosed pulmonary TB, (3) at least one positive sputum smear of acid fast bacilli, and (4) positive sputum culture of *Mycobacterium tuberculosis*. Patients with anti-tuberculosis drug-resistant strains were excluded. All patients were Japanese and had an academic background higher than junior high school graduation. Socio-demographic characteristics, including smoking history and alcohol drinking habits, were collected by nurses. Patients were considered to have a habit of alcohol drinking if they had ever drunk regularly for at least 1 year. Clinical, microbiological, radiological, and therapeutic data were collected from medical records.

### Anti-tuberculosis regimen

All patients were hospitalized, and most of them received standard treatment proposed by the Japanese Society for Tuberculosis [10] as soon as a positive sputum smear of acid fast bacilli was confirmed. During the initial phase, which lasted for 2 months, the patients took isoniazid (H), rifampicin (R), pyrazinamide (Z), and ethambutol (E) or streptomycin (SM) every day. During the continuation phase, which lasted for at least 4 months, the patients took H and R every day. If the patients could not tolerate pyrazinamide during the initial phase, the continuation phase would be extended to 7 months. The regimen was modified based on the presence of concomitant diseases or adverse effects. Directly Observed Treatment (DOT) was given by well-trained nurses during hospitalization. All patients were hospitalized for at least 2 months and discharged after the confirmation of sputum culture conversion.

### Sputum and radiographic evaluations

Sputum smear and culture examinations were performed on 3 consecutive days at the start of the treatment and then on 2 consecutive days every 2 weeks, with a margin of error of a few days, during the hospitalization. The sputum smears were examined using the Ziehl-Nielsen stain and graded according to the American Thoracic Society guidelines. [11] Sputum specimens were cultured on Ogawa’s medium.

The date of sputum culture conversion was defined as the date when the first 2 consecutive sputum specimens with a negative culture were taken, given that all subsequent sputum cultures were negative. Time to sputum culture conversion was calculated as the total days from the date of starting anti-tuberculosis treatment to the date of sputum culture conversion.

Patients’ CXRs were assessed by pulmonologists with the scoring system proposed by the Japanese Society for Tuberculosis. [12] The extent of the disease was graded from 1 to 3. Grade 1 was defined as minimal lung disease, in which the total area of the infiltrates was smaller than a lung portion extending from the horizontal line through the second rib up to the lung apex. Grade 3 was defined as advanced lung disease, in which the total area of the infiltrates was larger than one lung. Grade 2 was defined as moderately advanced disease, in which the total area of the infiltrates was between grades 1 and 3.

### Statistical analysis

Continuous variables are summarized as medians (interquartile range (IQR)), and categorical variables are summarized as frequencies and percentages. Cox proportional- hazards regression analysis was used to estimate the hazard ratio of sputum culture conversion for the following pre-specified baseline variables: age, sex, smoking history, drinking habit, diabetes mellitus, the presence of cavitation and the extent of the disease on CXR, and sputum smear grading. These baseline variables were chosen because they are considered clinically to affect the pathology of TB, and they have been reported as the factors that affect the time to sputum culture conversion in previous studies. The proportional hazards assumption was graphically verified using log-log plots.

Data were analyzed with the use of JMP software, version 9 (SAS Institute). A P-value < 0.05 was considered significant.

## Results

### Patients’ baseline characteristics

The database was composed of the data of 120 patients and 89 patients met the inclusion criteria.

Two patients with rifampicin-resistant strains and one patient with an isoniazid-resistant strain were excluded. The data of 86 patients were analyzed. The patients’ baseline characteristics are shown in Table 1. Their median age was 57 years; 70% were males; and 57% were smokers (current and ex-smokers). All smokers had a duration of smoking longer than three years. The majority of smokers were current smokers at the time of admission. However, all smokers stopped smoking after they were admitted to the hospital. The median pack-years in smokers was 40 (IQR, 20–53). Half of the patients (54.7%) had a habit of alcohol drinking. Nearly a third of the patients (31.4%) had a history of diabetes mellitus. No patients were positive for human immunodeficiency virus (HIV) antibody.

**Table 1:** Baseline characteristics of the 86 patients.

**Age (range)**		57 (40–67)
**Sex, n (%)**	Female	26 (30.2)
	Male	60 (69.8)
**Cigarette smoking, n (%)**	Current smoker	45 (52.3)
	Ex-smoker	4 (4.7)
	Non-smoker	37 (43.0)
**Drinking habit, n (%)**		47 (54.7)
**Diabetes mellitus, n (%)**		27 (31.4)
**Cancer, n (%)**		5 (5.8)
**Chest X-ray, n (%)**	Cavitation	58 (67.4)
	Extent* Grade 1	12 (14.0)
	Extent* Grade 2	61 (70.9)
	Extent* Grade 3	13 (15.1)
**Sputum smear grading, n (%)**	0	16 (18.6)
	Scanty positive	6 (7.0)
	1+	7 (8.1)
	2+	5 (5.8)
	3+	38 (44.2)
	4+	14 (16.3)
**Colony count, n (%)**	<10 colonies	16 (18.6)
	10–100 colonies	43 (50.0)
	>100 colonies	27 (31.4)

Data are presented as n (%) or median (IQR)

*See text for the definition of extent on chest X-ray.

Because this was a historical cohort study, there were some missing data related to the details of the therapeutic regimens. The initial therapeutic regimens of 85 patients were confirmed: 81 patients started therapy with the standard regimen and 4 patients with a modified regimen. Even if the regimen had been changed somewhat, it was confirmed that all patients were treated with multiple anti-tuberculosis drugs.

### Time to sputum culture conversion

The median duration of hospitalization was 119 days. No patients were lost to follow-up up to the end of the study. All patients had negative sputum cultures at the end of the study; there were no censored cases.

**Fig 1:**
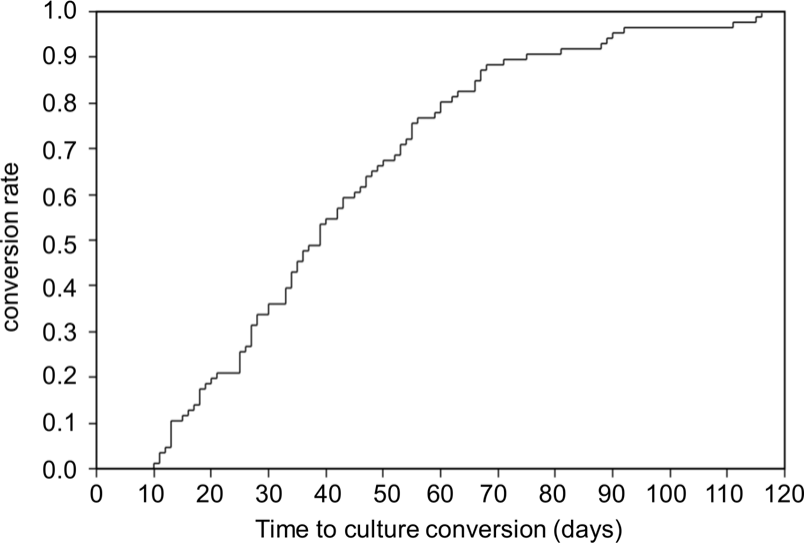
Kaplan-Meier Curve of the time to sputum culture conversion for all patients.

**Fig 2:**
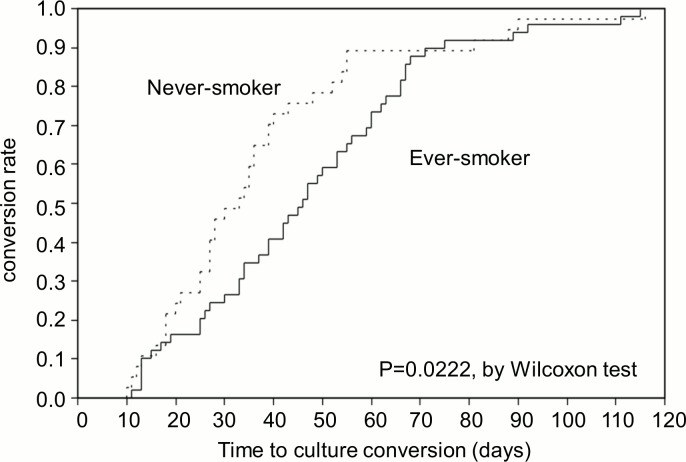
Kaplan-Meier Curves of the time to sputum culture conversion according to smoking history.

**Fig 3:**
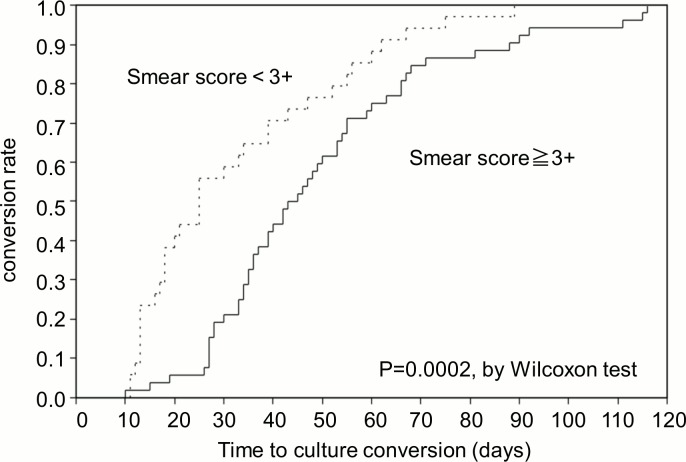
Kaplan-Meier Curves of the time to sputum culture conversion according to sputum smear grading.

The median time to sputum culture conversion was 39 days (IQR, 25–55), and the maximum time to sputum culture conversion was 116 days (Fig 1). The results of the Cox proportional-hazards regression analysis are shown in Table 2. A higher smear grading (grade 3+ or 4+) was associated with delayed culture conversion in the univariate model (HR, 0.51; 95%CI, 0.33–0.80). Although a history of smoking was not significant on univariate analysis, the confidence interval was suggestive of a delayed effect on conversion (HR, 0.70; 95%CI, 0.45–1.08). After adjusting for covariates, a history of smoking (HR, 0.48; 95%CI, 0.25–0.94) and a higher smear grading (HR, 0.40; 95%CI, 0.23–0.71) were associated with delayed culture conversion. In other words, at any time point during the treatment course, the odds of culture conversion were 52% less in smokers than in non-smokers and 60% less in patients with sputum smear grade ≥ 3+ than in those with smear grade < 3. The Kaplan-Meier curves showed that the conversion rate differed significantly between smokers and non-smokers mainly during the first 2 months of the treatment course (Fig 2). On the other hand, the conversion rate was significantly different between patients with high and low sputum smear grading constantly throughout the treatment course (Fig 3).

**Table 2:** Predictors of Time to Sputum Culture Conversion by Cox Proportional-Hazards Regression.

Predictors	Unadjusted Model		Adjusted Model	
	HR	95% CI	HR	95% CI
Age	1.00	0.99–1.01	1.00	0.98–1.00
Male sex	0.77	0.49–1.27	1.25	0.65–2.47
Diabetes mellitus	1.17	0.73–1.84	1.00	0.57–1.73
Smoker	0.70	0.45–1.08	0.48	0.25–0.94
Drinking habit	1.04	0.68–1.61	1.43	0.83–2.50
Extent/Chest X-ray				
1	reference		reference	
2	0.98	0.55–1.93	1.54	0.73–3.44
3	0.61	0.27–1.37	1.07	0.42–2.74
Cavity/Chest X-ray	0.86	0.54–1.37	1.11	0.65–1.95
Sputum smear grading≧3+	0.51	0.33–0.80	0.40	0.23–0.71

## Discussion

In the present study, a higher sputum smear grading and a history of smoking were associated with prolonged time to sputum culture conversion in adults with pulmonary tuberculosis.

Caetano Mota et al. reported that patients with a high pre-treatment colony count were less likely to convert than patients with a low pre-treatment colony count. [5] In the present study, although sputum smear grading, which is available at an early time point of treatment, was chosen as an index of bacillary load, the result is similar to that of the previous report. It may be natural that patients with a higher colony count take a longer time to convert sputum cultures. This result suggests the importance of early detection and rapid cure.

In a qualitative systematic review and meta-analysis, the evidence was rated as strong for an association between smoking and TB disease, moderate for the association between smoking and retreatment of TB disease, and limited for the association between smoking and TB infection and mortality. [13] While it has been reported that there was insufficient evidence to support the association between smoking and delayed sputum culture conversion, the result of the present study suggests that smoking is one of the factors that prolongs time to sputum culture conversion. Visser et al. also found that smokers had a significantly longer time to sputum culture conversion than non-smokers during the first 2 months of treatment. [4] Similarly, Maciel et al. reported that patients who smoked had three-fold greater odds of remaining sputum culture-positive after 2 months of treatment than non-smokers. [7] A prospective cohort study from Brazil showed that there was a dose-response relationship between the number of cigarettes smoked daily and the proportion of patients with positive cultures after 2 months of treatment. [8] Although these reports had comparatively small sample sizes, the recent large-sized cohort study in Hong Kong showed that ever-smokers had increased risks of persistently positive smears and cultures after 2 months of treatment compared with never-smokers. [9] On the other hand, there are conflicting data on the role of smoking in sputum culture conversion. [5,6] In the present study, all smokers stopped smoking after they were admitted to the hospital. Therefore, it can be inferred that smoking behavior before anti-TB treatment has a harmful effect on culture conversion. There are several reports that cigarette smoke weakens the immune response to tuberculosis. Shang et al. reported that cigarette smoke suppresses the protective immune response to *M*. *tuberculosis* in mice, human THP-1 cells, and primary human alveolar macrophages. [14] Shaler et al. reported that cigarette smoke exposure severely impeded the lung expression of anti-TB Th1 immunity via inhibiting innate immune activation and lung T cell recruitment. However, the effect on immune function disappeared with smoking cessation. [15] O'Leary et al. showed that alveolar macrophages (AMs) taken from ever-smokers failed to secrete significantly more cytokines after TB infection. [16] These reports may answer the question why smoking prolongs the time to sputum culture conversion. It might be inferred from the discussion so far that non-smoking from the beginning and early smoking cessation are important for shortening the time to sputum culture conversion.

The present study has several limitations. First, it might not have been possible to detect the association between CXR findings and time to sputum culture conversion because the sample size was small. Second, it was not possible to evaluate glycemic control. While diabetes mellitus was not a factor affecting the time to sputum culture conversion, patients were compared only on the basis of a past history of diabetes mellitus. Third, the amount of alcohol intake was not evaluated; it has been suggested that heavy drinking has more harmful effects than moderate drinking.

The present study, however, has the following strong points. First, because all the patients had been admitted to the hospital until sputum culture conversion, they were well controlled and received detailed follow-up. For example, all of them stopped smoking and received DOT every day. Therefore, it was possible to evaluate the effects of main factors including sputum smear grading and a history of smoking on time to sputum culture conversion throughout the course of tuberculosis treatment after controlling for other confounders. Second, because the sputum specimens of all patients were cultured every 2 weeks until sputum culture conversion, the percentage of patients with sputum culture conversion at certain time points, such as 2 weeks, 4 weeks, 6 weeks, and 8 weeks, could be determined. Third, this study involved a comparatively homogeneous population and controlled for the following confounders: race, academic background, and social stratification. Japan is unique amongst nations with good quality health care systems to have a comparatively high TB rate. However, the result may differ in a different population, and further studies with a larger sample size in different populations will be required to evaluate the effects of various factors on the time to sputum culture conversion.

In conclusion, a high sputum smear grading and a history of smoking were associated with prolongation of the time to sputum culture conversion in adults with pulmonary tuberculosis. A multidimensional approach will be required to effectively control TB, including early detection, timely anti-TB treatment, and appropriate interventions to lower the cigarette smoking rate.

## References

[1] World Health Organization. Fact sheet on tuberculosis. 2014; Available: http://www.who.int/mediacentre/factsheets/fs104/en/.

[2] Mitchison DA. Assessment of new sterilizing drugs for treating pulmonary tuberculosis by culture at 2 months. Am Rev Respir Dis. 1993;147(4):1062–3. 10.1164/ajrccm/147.4.1062 . 8466107

[3] Wallis RS, Doherty TM, Onyebujoh P, Vahedi M, Laang H, Olesen O, et al Biomarkers for tuberculosis disease activity, cure, and relapse. Lancet Infect Dis. 2009;9(3):162–72. 10.1016/S1473-3099(09)70042-8 . 19246020

[4] Visser ME, Stead MC, Walzl G, Warren R, Schomaker M, Grewal HM, et al Baseline predictors of sputum culture conversion in pulmonary tuberculosis: importance of cavities, smoking, time to detection and W-Beijing genotype. PLoS One. 2012;7(1):e29588 10.1371/journal.pone.0029588 22238625 PMC3251579

[5] Caetano Mota P, Carvalho A, Valente I, Braga R, Duarte R. Predictors of delayed sputum smear and culture conversion among a Portuguese population with pulmonary tuberculosis. Rev Port Pneumol. 2012;18(2):72–9. 10.1016/j.rppneu.2011.12.005 . 22277838

[6] Güler M, Unsal E, Dursun B, Aydln O, Capan N. Factors influencing sputum smear and culture conversion time among patients with new case pulmonary tuberculosis. Int J Clin Pract. 2007;61(2):231–5. 10.1111/j.1742-1241.2006.01131.x . 17166185

[7] Maciel EL, Brioschi AP, Peres RL, Guidoni LM, Ribeiro FK, Hadad DJ, et al Smoking and 2-month culture conversion during anti-tuberculosis treatment. Int J Tuberc Lung Dis. 2013;17(2):225–8. 10.5588/ijtld.12.0426 . 23317958 PMC4497564

[8] Nijenbandring de Boer R, Oliveira e Souza Filho JB, Cobelens F, Ramalho DeP, Campino Miranda PF, Logo K, et al Delayed culture conversion due to cigarette smoking in active pulmonary tuberculosis patients. Tuberculosis (Edinb). 2014;94(1):87–91. 10.1016/j.tube.2013.10.005 . 24321739

[9] Leung CC, Yew WW, Chan CK, Chang KC, Law WS, Lee SN, et al Smoking adversely affects treatment response, outcome and relapse in tuberculosis. Eur Respir J. 2015;45(3):738–45. 10.1183/09031936.00114214 . 25359352

[10] The Japanese Society for Tuberculosis. Manual of Medical Care Standards of Tuberculosis. Japan: Japan Anti-Tuberculosis Association; 1996

[11] Diagnostic Standards and Classification of Tuberculosis in Adults and Children. Am J Respir Crit Care Med. 2000;161(4 Pt 1):1376–95. 10.1164/ajrccm.161.4.16141 . 10764337

[12] The Committee for Classification of Tuberculosis in the Japanese Society for Tuberculosis. The criteria for chest roentgenogram classification of pulmonary tuberculosis established by the Japanese Society for Tuberculosis. Kekkaku. 1959; 34: 885

[13] Slama K, Chiang CY, Enarson DA, Hassmiller K, Fanning A, Gupta P, et al Tobacco and tuberculosis: a qualitative systematic review and meta-analysis. Int J Tuberc Lung Dis. 2007;11(10):1049–61. . 17945060

[14] Shang S, Ordway D, Henao-Tamayo M, Bai X, Oberley-Deegan R, Shanley C, et al Cigarette smoke increases susceptibility to tuberculosis—evidence from in vivo and in vitro models. J Infect Dis. 2011;203(9):1240–8. 10.1093/infdis/jir009 . 21357942

[15] Shaler CR, Horvath CN, McCormick S, Jeyanathan M, Khera A, Zganiacz A, et al Continuous and discontinuous cigarette smoke exposure differentially affects protective Th1 immunity against pulmonary tuberculosis. PLoS One. 2013;8(3):e59185 10.1371/journal.pone.0059185 23527127 PMC3602464

[16] O'Leary SM, Coleman MM, Chew WM, Morrow C, McLaughlin AM, Gleeson LE, et al Cigarette smoking impairs human pulmonary immunity to Mycobacterium tuberculosis. Am J Respir Crit Care Med. 2014;190(12):1430–6. 10.1164/rccm.201407-1385OC . 25390734

